# Recent Developments in Mendelian Randomization Studies

**DOI:** 10.1007/s40471-017-0128-6

**Published:** 2017-11-22

**Authors:** Jie Zheng, Denis Baird, Maria-Carolina Borges, Jack Bowden, Gibran Hemani, Philip Haycock, David M. Evans, George Davey Smith

**Affiliations:** 1 MRC Integrative Epidemiology Unit, University of Bristol, Oakfield House, Bristol, UK; 2 University of Queensland Diamantina Institute, Translational Research Institute, University of Queensland, Brisbane, QLD Australia

**Keywords:** Mendelian randomization, Databases and automation tools for causal inference, Hypothesis-free causality, Drug development, Disease progression

## Abstract

**Purpose of Review:**

Mendelian randomization (MR) is a strategy for evaluating causality in observational epidemiological studies. MR exploits the fact that genotypes are not generally susceptible to reverse causation and confounding, due to their fixed nature and Mendel’s First and Second Laws of Inheritance. MR has the potential to provide information on causality in many situations where randomized controlled trials are not possible, but the results of MR studies must be interpreted carefully to avoid drawing erroneous conclusions.

**Recent Findings:**

In this review, we outline the principles behind MR, as well as assumptions and limitations of the method. Extensions to the basic approach are discussed, including two-sample MR, bidirectional MR, two-step MR, multivariable MR, and factorial MR. We also consider some new applications and recent developments in the methodology, including its ability to inform drug development, automation of the method using tools such as MR-Base, and phenome-wide and hypothesis-free MR.

**Summary:**

In conjunction with the growing availability of large-scale genomic databases, higher level of automation and increased robustness of the methods, MR promises to be a valuable strategy to examine causality in complex biological/omics networks, inform drug development and prioritize intervention targets for disease prevention in the future.

## Introduction

**Fig. 1:**
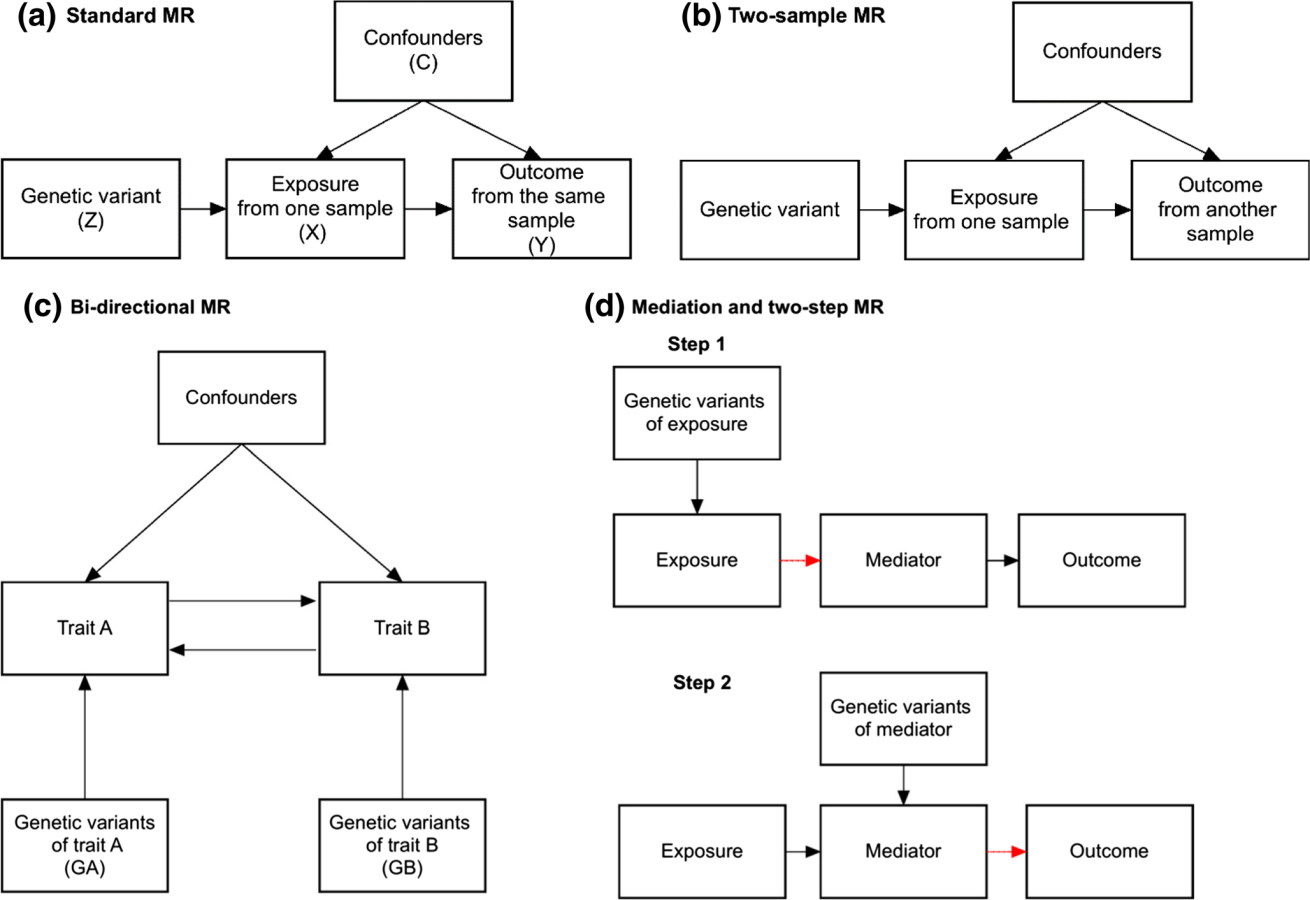

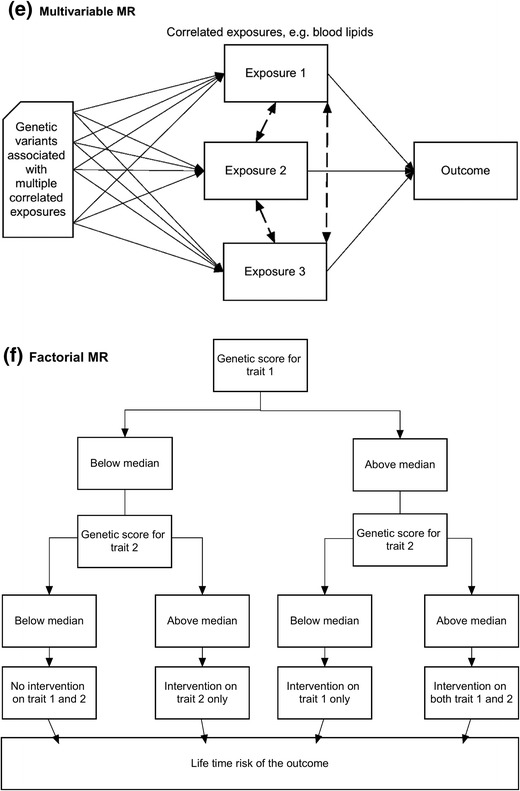
Design strategies for Mendelian randomization. **a** Standard MR: The causal relationship between an exposure variable (X) and an outcome (Y) is estimated using genetic variants (Z) as an instrument, regardless of the presence of variables (C) that may confound the observational association between the exposure and outcome. One method of estimation involves calculation of the Wald Ratio, [see Burgess review paper for description of the various instrumental variable (IV) estimators available] [3], where the causal estimate (${\widehat{\beta}}_{IV}$) is derived by dividing the estimated regression coefficient of the outcome on the single nucleotide polymorphism (SNP) (${\widehat{\beta}}_{YZ}$) by the estimated regression coefficient of the exposure on the SNP (${\widehat{\beta}}_{XZ}$). **b** Two-sample MR. **c** Bidirectional MR. **d** Mediation and two-step MR. **e** Multivariable MR. **f** Factorial MR

Causal inference in traditional observational epidemiological studies is hampered by the possibility of confounding and reserve causation [1]. Mendelian randomization (MR) is a method that can be used to uncover casual relationships between an exposure and outcome in the presence of such limitations. MR is a form of instrumental variable analysis, where genetic variants are used as proxies for the exposure of interest [2]. As Mendel’s Laws of Inheritance dictate, alleles segregate randomly from parents to offspring. Thus, offspring genotypes are unlikely to be associated with confounders in the population. In addition, germ-line genotypes are fixed at conception, and therefore, temporally precede the variables under observation, avoiding issues of reverse causation. The MR method involves finding genetic variants which are associated with an exposure, and then testing the association between these variants and the outcome. The causal “de-confounded” relationship between exposure and outcome can then be estimated when the necessary conditions are satisfied (Fig. 1a).

In understanding how MR works, it can be useful to think of an MR study as being analogous to a randomized controlled trial (RCT), except that genotypes are used to randomize participants into different levels of the exposure/treatment. However, it is important to realize that this analogy is not perfect e.g., RCTs typically involve treatments over a short duration, whereas an individual’s genetics influences their biology from conception, meaning that many causal estimates from MR studies might reflect life-long exposures as well as developmental compensation that may arise from inheriting these mutations [4••].

Although initial applications of MR mostly focused on estimating the causal effect of environmental exposures on medically relevant outcomes, in recent years MR has found utility across a wide range of domains including the development of pharmaceutical agents (i.e., drug target validation, drug target repurposing, and side effect identification) and in the interpretation of high-dimensional omics studies. Table 1 lists several recent studies illustrating how MR has been used successfully across a wide variety of different contexts [5–37•, 106].

**Table 1:** Recent Mendelian randomization studies

Type	Exposure	Outcome	Importance	References
Drug target validation	*HMGCR* (statins)	Metabolites	MR study suggests that genetic polymorphisms in the *HMGCR* gene mimic the effect of statins on a wide range of metabolomic measures	[5•]
Drug target validation	*NPC1L1* (ezetimibe)	CHD	MR study suggests that lowering LDL cholesterol level via inhibition of *NPC1L1* results in reduced risk of CHD consistent with results from RCTs of the drug ezetimibe which targets this pathway	[6•, 7]
Drug target validation	PCSK9 (evolucumab)	CHD	Genetic evidence suggests that polymorphisms in the *PCSK9* gene are strongly related to LDL cholesterol levels and risk of CHD. Randomized controlled trials have shown that PCSK9 inhibition is associated with decreased LDL cholesterol levels and risk of myocardial infarction in hyperlipidemic individuals. Several agents that target PCSK9 are now FDA approved for the treatment of hyperlipidemia including the monoclonal antibodies evolucumab and alirocumab. Antisense oligonucleotides that target PCSK9 are also in development	[8•, 9]
Drug target validation	CRP	CHD	Despite serum levels of C-reactive protein having marked clinical utility as a biomarker of inflammation, MR studies have consistently failed to find evidence for a causal effect of serum C-reactive protein on risk of cardio-metabolic disease. These MR studies have potentially saved pharmaceutical companies from developing agents that would be destined to fail in RCTs	[10, 11]
Drug target validation	Lp-PLA2 (darapladib)	CHD	Many resources were spent on trials that showed therapeutic lowering of Lp-PLA2 level does not lower risk of CVD; some MR studies were published before the reporting of RCT results	[12, 13]
Drug target repurposing	IL6 (tocilizumab)	CHD	Repurposing blockade of the interleukin-6 receptor (tocilizumab) as therapeutic approach to prevention of coronary heart disease	[14•]
Predicting side effects of drug targets	*HMGCR* (statins)	Type 2 diabetes	MR studies indicated that the increased incidence of type 2 diabetes among statin users in RCTs was likely to be due to an on-target effect of statins on *HMGCR* inhibition	[15•]
Predicting side effects of drug targets	*PCSK9/PCSK9* inhibitors	Type 2 diabetes	Suggests *PCSK9* inhibition might increase risk of diabetes	[16, 17]
Public health/clinical practice	Adiposity (BMI and waist–hip ratio)	CHD	Suggests adiposity causes CHD, heart failure and ischemic stroke. No trial has yet shown this causal relationship	[18, 19]
Public health/clinical practice	LDL cholesterol	Diabetes	Suggests LDL cholesterol lowering might generally lead to increased risk of diabetes mellitus, and has potential ramifications for drugs that lower LDL cholesterol level	[20]
Public health/clinical practice	Triglycerides	CHD	MR studies suggest that lowering triglycerides will reduce risk of CHD.	[20, 21•, 22]
Public health/clinical practice	Educational attainment	CHD	MR findings suggest that policy interventions that increase education may reduce the burden of cardiovascular disease	[23]
Public health/clinical practice	Vitamin D	Multiple Sclerosis	MR studies suggest that lowered vitamin D level is causally associated with increased susceptibility to MS.	[24, 25]
Public health/clinical practice	Alcohol	CVDs (including blood pressure, coronary artery calcification, and CHD)	Suggests alcohol is harmful to cardiovascular health at all doses of consumption, contrary to decades of observational data	[26, 27]
Public health/clinical practice	Telomere length	Cancers, cardiovascular diseases, and other diseases	Large-scale MR study suggests that longer telomeres increase risk for some cancers but reduce risk for some non-neoplastic diseases, including cardiovascular diseases, which highlight the value of MR at a phenome wide scale	[28•]
Public health/clinical practice	Obesity, type 2 diabetes, Metabolic factors	Pancreatic Cancer	MR study suggests a causal role of BMI and fasting insulin in pancreatic cancer etiology	[29]
Public health/clinical practice	Blood lipids	Prostate cancer	MR study shows evidence that higher LDL-C and triglycerides levels increase aggressive prostate cancer risk	[30]
Public health/clinical practice	Childhood adiposity	Type 1 diabetes	MR study provides genetic evidence for childhood adiposity as a risk factor for Type 1 diabetes	[32]
Public health/clinical	Vitamin D	Cancer	MR study suggests that vitamin D supplementation should not currently be recommended as a strategy for primary cancer prevention.	[31]
Public health/clinical practice	Cannabis use	Schizophrenia	MR study suggests cannabis use to be associated with increased risk of schizophrenia	[106]
Complex molecular traits	Blood cell traits	Complex human traits	Large-scale MR as a follow-up of GWAS of complex molecular traits, which suggests causal relationship between blood cell indices and autoimmune diseases, schizophrenia, and coronary heart disease	[34•]
Complex molecular traits	Methylation QTLs	Cardiovascular and complex traits	Systematically explores the genetic influences on complex disease mediated by DNA methylation using MR and fine mapping. MR suggests causal relationships between methylation levels and 14 cardiovascular disease traits	[35]
Complex molecular traits	Expression QTLs	Complex human traits	Integrated expression QTLs with summary GWAS results of human diseases using SMR (more details of the method in Table 2), which prioritized 126 genes associated with 5 complex diseases and highlights the importance of considering horizontal pleiotropy in MR	[36]
Complex molecular traits	Protein QTLs	Vascular, neoplastic, and autoimmune diseases	Identified causal roles for protein biomarkers in disease, which suggests a causal relationship for IL1RL1-IL18R1 loci on atopic dermatitis as well as MMP-12 on CHD	[37•]

## Core Assumptions Underlying Causal Inference in Mendelian Randomization Studies

In order for a genetic variant to qualify as a valid instrument for causal inference in a MR study, it must satisfy three core assumptions:

- Assumption 1: The genetic variant must be truly associated with the exposure (NB the SNP need not be the functional variant responsible for the SNP-exposure association). Typically, SNPs which pass genome-wide significance (*P* < 5 × 10^−8^) and have been replicated in an independent sample are used as instruments in MR studies. The use of weak instruments can bias MR estimates towards the confounded observational estimate in one-sample MR settings and towards the null in two-sample MR settings (with non-overlapping samples). As common genetic variants frequently explain a small proportion of a trait’s variance, it may be useful to combine the effects of many SNPs together in an allelic score and use this as an instrument in MR studies.
- Assumption 2: The genetic variant should not be associated with confounders of the exposure-outcome relationship. Although it is technically impossible to prove that this assumption holds in a MR study, it may be possible to disprove it by examining the association between the variant and known confounders of the exposure-outcome relationship.
- Assumption 3: The genetic variant should only be related to the outcome of interest through the exposure under study. This is commonly referred to as the “no pleiotropy” assumption or the exclusion restriction criterion. Horizontal pleiotropy, where a SNP is associated with multiple traits independently of the exposure of interest, potentially violates this assumption. While it is not possible to prove that this assumption holds in an MR study, various extensions of the basic MR design can be used to detect its presence, and estimate the causal effect of the exposure even in the presence of such violation of the assumption (see below).

Even when these core assumptions have been met, MR has a number of limitations which need to be considered (summarized in Table 2), and which have been discussed at length elsewhere [2, 45–49, 50•, 51•, 52•].

**Table 2:** Potential pitfalls in the interpretation of MR Studies and suggestions for dealing with these

Limitation	Description	Solution
Weak instrument bias	Weakly associated variants (F statistics < 10) can bias causal estimates towards the null for two-sample MR and towards the observational estimate for one-sample MR	Increase sample sizes through utilizing large publicly available GWAS datasets (e.g., UK Biobank) or summary GWAS results data. Use of allele scores explaining more variation in the exposure
Lack of reliable genetic instruments for exposure of interest	Genetic instruments are not available for some exposures	Conduct MR on a similar exposure (proxy phenotype) for which GWAS data is available, for example, BMI is often used as a proxy of overall adiposity [18, 19]. Polygenic score (PGS) approaches can be used when there is a lack of reliable instruments [38•, 39–41], although the results of these analyses must be interpreted cautiously due to concerns about their lack of specificity and possible reintroduction of pleiotropy
Population stratification	Spurious associations may arise in MR where the genetic variant and the outcome are associated with ancestral background in an admixed or stratified sample	Use genetic associations derived from within homogenous populations only. Use summary results statistics from GWAS that have adequately controlled for population substructure through e.g. principal components analysis or linear mixed models
Low power	Causal estimates are imprecise (wide confidence intervals) and the MR analysis lacks power to detect a causal effect (1 – probability (type II error)). Power is a function of sample size, variance explained in the exposure by the SNP, causal effect size, strength of confounding, and type 1 error rate. Approximate power can be determined using a freely available web application [33]	Same as above for weak instruments
Horizontal pleiotropy	The genetic instrument is associated with the outcome via pathway that does not pass through the exposure of interest [20]	Better understanding of the underlying biological function of genetic variants/genes (for example using selected candidate loci). Utilizing variants which directly code for the exposure of interest (e.g., variants in the C-reactive protein gene to proxy serum levels of C-reactive protein). Specialized methods for providing estimates robust to horizontal pleiotropy with some relaxation of the IV assumptions (e.g., MR-Egger regression [42••]; Weighted Median approach [43•]), and for detecting pleiotropy, are described in Table 4
Linkage disequilibrium (LD)	Confounding can be re-introduced in the analysis through a variant in LD with the instrument that exerts an effect on the outcome through a pathway other than through the exposure of interest	As above for pleiotropy
Canalization/developmental compensation	An individual adapts in response to a genetic change so that the effect of that genetic change is reduced or absent. MR may produce causal estimates that are not representative of effects that would be produced by modifying the exposure	Extent of the impact of canalization on MR is currently unclear. Greater understanding of the patterns of gene expression/regulation during development is required to evaluate the plausibility and consequences of canalization
Complexity of biology	Due to the underlying complexity of biological pathways overly simplistic interpretations can be misleading. For example, a genetic variant in the IL-6 receptor (*IL6R*) leads to reduced membrane-bound *IL6*, which in turn results in increased levels of circulating *IL6*, disruption of classical *IL6* signaling, reduced C-reactive protein (CRP) levels, and a reduction in risk of CHD. This process should not be naively interpreted as circulatory increasing *IL6* as being is protective against CHD [4••]	Improved understanding of underlying molecular biology and exact pathways involved
Winner’s curse	In the case of single-sample MR, utilizing the same sample as a discovery analysis for genetic instruments is not a good idea because estimates of the SNP-exposure association will be biased upwards. In the case of two-sample MR, genetic associations published in discovery GWAS may overestimate the SNP-trait association, particularly if the GWAS is underpowered to detect the particular loci. In the case of GWAS of exposures, this will overestimate the effect of the genetic instrument relative to the exposure and result in bias of the causal estimate towards the null. Likewise, in the case of GWAS of outcomes, winner’s curse will overestimate the association between the genetic instrument and the outcome and lead to a bias in causal estimates away from the null	In the case of single-sample MR, using an unweighted allelic score of several variants may provide a sensitivity analysis. In the case of two-sample MR, utilize estimates from the replication analysis if these are appropriately precise
Trait heterogeneity	Genetic instruments are sometimes associated with multiple aspects of traits (exposures). Such heterogeneity does not preclude causal inference but it does undermine the ability to infer causality for particular dimensions of heterogeneous exposures and makes interpretation of MR analyses more difficult	Only biological knowledge was able to resolve the particular dimension of the biological pathway causally relevant to certain outcomes (e.g., diseases). Further research is required in this area
Collider bias	Collider bias occurs when the exposure and outcome of interest independently influence a third risk factor, and this third risk factor is conditioned upon. Collider bias is mostly likely to occur in MR studies which are influenced by high attrition rates (loss to follow up), in case only studies (disease progression), and in MR studies where the instruments are generated in a GWAS which conditions one phenotype on another (e.g. waist circumference on CHD adjusted for BMI) [44•]	Given the influence of selection and attrition on a study is known, this could lead to biased estimates of both phenotype and genetic association. For example, having DNA available on most participants in a birth cohort study offers the possibility of investigating the extent to which polygenic scores predict subsequent participation, which in turn would enable sensitivity analyses of the extent to which bias might distort estimates

## Design of Mendelian Randomization Studies

The term MR covers a variety of approaches that use genetic variants to make inferences about the causal relationship between traits of interest [45, 52•]. Figure 1 illustrates some extensions to the basic MR design which are described in more detail in the paragraphs below.

### Two-Sample Mendelian Randomization

Prior to 2011, most MR analyses were conducted using genetic instruments, exposure, and outcome of interest from individuals measured in the same sample (this is termed one-sample MR or single-sample MR). In such a scenario, the causal effect of the exposure on the outcome was typically estimated using 2-stage least-squares (2SLS) regression [53] (Fig. 1a). However, it is also possible to use MR to estimate causal effects where data on the exposure and outcome have been measured in different (or only partially overlapping) samples. This is known as two-sample MR [54•] (Fig. 1b). There are many advantages of using two-sample MR including in situations where it is difficult and/or expensive to measure the exposure and outcome in the same set of individuals (e.g., studies involving molecular gene expression data). Two-sample MR greatly increases the scope of MR analysis and continues to grow in popularity. For example, two-sample MR analyses can be performed on publicly available genome-wide association study (GWAS) summary data, a fact that has been taken advantage of by web software (and R packages) like MR-Base [55••]. Two-sample MR is understandably becoming increasingly popular in the research community. The percentage of all MR studies that used the two-sample design framework rose from close to 0% in 2011 to around 40% in 2016 [56].

### Bidirectional Mendelian Randomization

In bidirectional MR, instruments for both exposure and outcome are used to evaluate whether the “exposure” variable causes the “outcome” or whether the “outcome” variable causes the “exposure” (Fig. 1c) [57•]. For example, in explaining the observational relationship between low levels of LDL cholesterol and risk of cancer, it may not be clear whether low levels of LDL cholesterol are causal for cancer, whether the presence of (undetected) cancer has a negative effect on LDL cholesterol, or whether the correlation between the two is due to latent confounding [58]. Bidirectional MR can help tease apart these relationships. MR analysis is first performed in one direction (i.e., “exposure” to “outcome”), and then performed in the opposite direction (i.e., “outcome” to “exposure”) using the SNPs robustly associated with each trait in the separate GWASs. The approach assumes that the causal association works through an underlying mechanism where it is possible to determine a single causal temporal direction. However, the complexity of biological systems, such as the existence of feedback loops between exposure and outcome variables, may make interpretation of the results of such analyses difficult [52•]. In these situations, it may be possible to use structural equation modeling to estimate feedback loops, although the properties of such approaches have yet to be examined thoroughly [51•].

### Two-Step Mendelian Randomization

Two-step MR is used to assess whether an intermediate trait acts as a causal mediator between an exposure and an outcome [59•]. As shown in Fig. 1d, in the first step of the procedure, genetic instruments for the exposure are used to estimate the causal effect of the exposure variable on the potential mediator. In the second step of the procedure, genetic instruments for the potential mediator are used to assess the causal effect of the mediator on the outcome. Evidence of association in both steps implies some degree of mediation of the association between the exposure and the outcome by the intermediate variable. The magnitude of the direct effect (which is the effect of exposure on the outcome independent of the mediator) and indirect effect (which is the effect of the exposure on the outcome via the mediator) can be estimated separately by this method [60•]. However, this does require the assumptions of linearity and homogeneity for both the exposure-mediator and exposure-outcome relationships and no statistical interaction between exposure and mediator [60•]. Two-step MR and two-sample MR can be combined to facilitate the investigation of causal mediation in very large samples of individuals [50•].

### Multivariable Mendelian Randomization

In some situations, genetic variants are pleiotropically associated with multiple correlated phenotypes. For example, genetic variants associated with lipoprotein metabolism rarely correlate with only one specific lipid fraction [61, 62•]. Single variable MR is likely to result in misleading conclusions regarding causality due to the presence of this horizontal pleiotropy. Multivariable MR is able to overcome this problem by using instruments associated with multiple exposures to jointly estimate the independent causal effect of each of the risk factors on the outcome (Fig. 1e) [63•, 64, 65]. For example, multivariable MR has recently been successfully employed in examining the relationship between high-density lipoprotein cholesterol and coronary heart disease. Univariate MR analyses, which ignore potential pleiotropic effects from other lipid fractions, suggest that increasing HDL levels lowers the risk of coronary heart disease. However, multivariable MR, which is able to account for SNPs’ pleiotropic effects through low-density lipoprotein and triglyceride levels [21•, 22], indicates that HDL is not causal for coronary heart disease, consistent with much of the evidence from randomized controlled trials [66–68].

### Factorial Mendelian Randomization

The manner by which causes of disease act together to increase disease risk can have important public health implications, as above-additive effects act together to generate a greater burden of disease in the population [69]. Factorial MR can be used to determine the combined causal effects of the co-occurrence of two or more risk factors for disease [6•, 45] (Fig. 1f). In order to conduct factorial MR, individual level genotype data are required. For example, Ference et al. conducted a factorial MR study in order to investigate the effects of HMGCR and PCSK9 inhibition on CHD risk. In this study, a weighted genetic score for PCKS9 inhibition was constructed (with the weighting based on each SNP’s effect on LDL cholesterol levels) and participants were allocated into either a high or low inhibition group based on the median value of the PCSK9 score. The genetic score for HMGCR inhibition was constructed and the individuals were further allocated into groups based on the median value of the HMGCR score (Fig. 1f). The causal estimates for PCSK9 and HMGCR inhibition, and the combined effect of the two on CHD could then be determined. Results from this factorial analysis suggested that HMGCR and PCSK9 inhibition have independent effects on CHD, and act together in an additive manner to reduce CHD risk [16]. Another example of a factorial MR suggested that *CETP* inhibitors and statins were associated with decreased LDL-C and apoB levels and reduced risk of cardiovascular events. The reduction in CVD risk was proportional to the apoB reduction but less than expected for the LDL-C reduction [70].

## Recent Developments

### Resources for Performing Mendelian Randomization Analyses

MR, and in particular two-sample MR, provides a powerful, cost-efficient, and simple way to investigate potential causal relationships between many different human traits. Usefully, many GWAS consortia have made the results of their meta-analyses publicly available, greatly facilitating the running of such analyses [71–73]. For example, as a centralized GWAS data resource, Phenoscanner [74], can be used to search for genetic association across a large number of phenotypes. In addition, Ben Neale’s group have recently provided GWAS results of more than 2400 human traits based on up to 337,000 individuals from the latest UK Biobank release enabling two-sample MR analyses on a very large number of individuals (data can be downloaded from http://www.nealelab.is/blog/2017/7/19/rapid-gwas-of-thousands-of-phenotypes-for-337000-samples-in-the-uk-biobank). Several large-scale biobanks, such as the UK Biobank [75], the China Kadoorie biobank [76], and the HUNT study [77] allow researchers to apply for (a certain level of) genotype and phenotype information on large numbers of participants. These data sources can be used in one- or two-sample MR analyses when combined with other datasets. This idea led to the development of MR-Base [55••], which retrospectively collected, harmonized, and centralized complete GWAS summary datasets from the public domain. The curated summary data corresponds to 135 diseases, almost 2000 phenotypes in 1.5 million individuals and up to 4 billion SNP-trait associations, which is integrated with a software infrastructure (web interface, R package and API) for automating MR analyses. Therefore, MR-Base greatly increases the accessibility of GWAS summary results to other researchers, accelerates identification (discovery strand), prioritization (evidence synthesis strand), and evaluation (translational strand) of intervention targets.

### Hypothesis-Free Investigations and “Mining the Phenome”

While there is obvious value in using MR to investigate the relationship between phenotypes for which causality has already been hypothesized, there is also an interest in detecting novel causal relationships. Hypothesis-free study designs such as genome-wide association studies (GWAS) and epigenome-wide association studies (EWAS) have shown tremendous success in recent years, and there are some instances where this strategy has shown promise in detecting putative causal relationships between phenotypes [38•, 51•, 78].

In a recent “one exposure to many outcomes” MR application, Haycock et al. systematically examined the association between telomere length and 22 cancers and 32 primary non-neoplastic diseases. The results suggested that longer telomeres were generally associated with increased risk for site-specific cancers but reduced risk for some non-neoplastic diseases, including cardiovascular diseases. This study highlighted the power of hypothesis-free MR in building a phenome-wide picture of traits of interest as opposed to the traditional “one exposure to one outcome” MR approach [28•].

Automation and data repositories provide solutions to some of the challenges involved in hypothesis-free MR. They trivialize the process of performing the analysis itself, and go some way towards improving reliability by (a) reducing human error [56] and (b) promoting the use of appropriate sensitivity analyses [3, 42••, 43•, 79, 80•, 81•]. However, many challenges still remain. Statistical power in MR is an issue even in the hypothesis-driven case, but hypothesis-free MR comes with a multiple testing burden that may be highly problematic. The nature of the data used in hypothesis-free MR is quite different from other hypothesis-driven study designs. There is often only a single consortium providing summary data for any one disease or trait which means that replication of a putative association in independent samples can be impossible. The emergence of large biobanks [75] may go some way to avoid this problem for many complex traits, but specific diseases for which cases need to be ascertained will still pose a challenge.

Another practical issue surrounds selecting those results from a hypothesis-free scan that are worthy of follow-up. Horizontal pleiotropy can manifest in many different patterns, which means that knowing the appropriate MR method to use for any particular pair of traits is difficult. Relying on a single method could lead to missed associations through of being overly conservative when there is no pleiotropy, or result in too many false positives because of miss-specifying the pleiotropic model. One method that has been developed recently to address this issue is MR-MoE (MR mixture of experts), which seeks to predict the most appropriate model based on the characteristics of the summary data [82•].

Another potential analytical strategy to mine the phenome would be to screen large publicly available disease and multi-omic GWAS summary results for evidence of genetic correlation using LD score regression via LD hub [83••, 84•]. Here, if traits are causally related and have non-zero heritability then there should be non-zero genetic correlations. However, genetic correlations can arise due to genetic confounding and horizontal pleiotropy and do not provide evidence on the direction of causality. Those disease-omic pairs showing evidence of genetic correlation could be followed up by conducting formal MR analyses [85]. One potential drawback of this approach is that the statistical efficiency of LD score regression may not be as high as that of MR in many cases, so selecting the appropriate scenarios in which to apply this as a screening method is important and warrants a *priori* power calculations.

### The Role of MR in Disease Progression and Treatment

To date, the large majority of GWAS identify genetic variants (SNPs) associated with incidence or risk of disease. Such variants are informative for disease prevention, but not necessarily for treatment aimed at influencing disease progression [86, 87•]. For example, only ~ 8% of genetic association hits in the GWAS Catalog (*p* < 1 × 10^−5^) were reported by studies that have attempted to identify variants associated with disease progression or severity, and most of these GWAS have limited statistical power owing to small sample sizes (90% have *N* < 5000) [87•]. In a systematic search of the literature, Paternoster et al. were able to identify only 27 genetic studies that have used MR to identify risk factors influencing disease progression [87•], which leaves massive scope to extend MR methodologies and applications in this area. The introduction of collider bias when studying a selected (e.g., case only) group of individuals [87•] is a particular challenge when studying disease progression (more details of collider bias are given in Table 3 and 4) [44•, 104].

**Table 3:** Databases and bioinformatic toolkits for performing MR

Name	Note	Web link	Ref
MR-Base	GWAS summary database of more than 1100 GWAS trails and online platform to automate MR	http://www.mrbase.org/	[55••]
MR-PRESSO	R package that allows for the evaluation of pleiotropy in multi-instrument Mendelian randomization	https://github.com/rondolab/MR-PRESSO	[88•]
Two-sample MR	R package for MR analysis, directly links to MR-Base database via API	https://github.com/MRCIEU/TwoSampleMR/	[55••]
Mendelian randomization	R package for MR analysis, links to Phenoscanner database	https://cran.r-project.org/web/packages/MendelianRandomization/	[89]
MR robust	STATA package for MR analysis	https://github.com/remlapmot/mrrobust/	[105]
Summary-data-based Mendelian randomization (SMR)	Linux package for MR analysis for testing expression QTL on complex diseases	http://cnsgenomics.com/software/smr/	[36•]
PHESANT	R package for performing phenome scans in UK Biobank, including MR phenome-wide association studies (MR-pheWAS)	https://github.com/MRCIEU/PHESANT/	[91•, 92]
PhenoSpD	R scripts to estimate multiple testing correction for hypothesis free MR	https://github.com/MRCIEU/PhenoSpD/	[93]

**Table 4:** Methods for dealing with limitations of MR

Category	Method	Description	Reference
Estimation of causal effect	Inverse variance weighted	Traditional MR method which uses a meta-analysis approach to combine the Wald ratio estimates of the causal effect obtained from different SNPs. The point estimates obtained from IVW MR are equivalent to a weighted linear regression of SNP-outcome associations on SNP-exposure associations with the intercept constrained to zero	[2, 79]
	MR-Egger	Unlike IVW, MR-Egger regression is not constrained to have a slope through zero, therefore its causal estimate represents a genotype-outcome dose response relationship which takes pleiotropic effects into account. It requires the InSIDE assumption to hold, which means the strength of the gene-exposure association should not correlate with the strength of bias due to pleiotropy	[42••]
	Weighted median	Defined as the median of a weighted empirical density function of the ratio estimates. Can consistently estimate the causal effect if at least 50% of the information in the analysis comes from valid instruments	[94•]
	Mode-based estimate (MBE)	The MBE provides a consistent estimate of the causal effect if the most common pleiotropy value across instruments is zero. This is termed the Zero Modal Pleiotropy Assumption (ZEMPA)	[80•]
	Pleiotropy-robust MR (PPMR)	Provides an unbiased causal estimate in the presence of pleiotropy, by subtracting the pleiotropic effect (of an instrument) estimated in a subgroup of the population for whom the instrument is not associated with the exposure. It assumes that such a sub-population exists, and the measure of genetic pleiotropy in this sub-population is the same as the general population	[27, 95]
	Generalized gene-environment interaction models	These methods provide unbiased estimates of the causal effect in the presence of horizontal pleiotropy by using the gene-environment interaction term in a linear interaction model as a valid instrument. It requires there exists variation in the strength of gene-exposure association across subgroups of the environmental covariate	[90, 96, 97]
	Likelihood-base methods	Assumes a linear relationship between the exposure and outcome and a bivariate normal distribution for the genetic estimates used. Naturally incorporate uncertainty and correlation between SNP-exposure and SNP-outcome parameter estimates. Yield efficient estimates when its specific modelling assumptions are met (and is therefore robust to weak instrument bias in this case), but is sensitive to model misspecification (for example due to invalid instruments).	[3, 79]
	Bayesian model averaging	Assumes prior probabilities for three pleiotropy models: (1) no pleiotropy; (2) pleiotropy with average value zero satisfying the InSIDE assumption; (3) non zero average pleiotropy satisfying the InSIDE assumption. Bayesian model averaging is then used for inference	[81•]
Testing for pleiotropy	MR-Egger intercept	Intercept of the MR-Egger regression captures the average pleiotropic effect across all genetic variants	[42••]
	Cochran Q (IVW), Rucker’s Q (MR-Egger)	Measure of heterogeneity between genetic instruments used which could indicate pleiotropic effects	[98–100]
	Cook’s distance/studentized residuals	Standard regression diagnostics that can be used to detect outliers that may distort the causal effect estimation	[101]
	Leave-one-out analysis	Systematic removal of genetic instruments from MR analysis to identify influential outliers	[55••]
Assessment of instrument strength	Mean F-statistic (IVW)	Used to measure the strength of genetic instruments in IVW and hence assess the influence of weak instrument bias. F < 10 is considered problematic	[102]
	I^2^ (MR-Egger)	Adaption of the I^2^ heterogeneity statistic in meta-analysis which measures the degree of regression dilution bias in the MR-Egger estimate in the two-sample setting	[43•]
Data visualization	Funnel plot	Plot of instrument strength versus causal effect estimate. Used to detect evidence of directional pleiotropy in MR-Egger analyses	[42••]
	Scatter plot	Scatter of genotype-outcome associations versus genotype-exposure associations. Used to detect departures from MR assumptions, and compare regression slopes from different MR analysis	[94•]
	Radial plot	Plots square root instrument strength times the causal estimate on the vertical axis versus the square root instrument strength on the horizontal axis. Facilitates a more straightforward detection of outliers in an IVW and MR-Egger analysis. It can also be used as a basis for a generalized form of MR-Egger regression “radial MR-Egger”	[99]
	Q-contribution plots	Depicts the contribution of individual genetic variants towards the overall heterogeneity observed in Cochran’s Q statistic (for IVW) and Rucker’s Q’ statistic (for MR-Egger). Used to identify pleiotropic variants by assessing each SNP’s contribution against a chi-squared distribution on 1 degree of freedom	[103]
	Forest plot	Compares the MR estimates for each genetic instrument to detect pleiotropic effects	[55••]

### The Development of Approaches to Detect and Correct for Horizontal Pleiotropy in MR Analysis

The possibility of horizontal pleiotropy and the consequent violation of the exclusion restriction criterion are widely seen as the greatest threat to the validity of MR studies. Over the last few years, investigators have developed a suite of approaches that relax the strict requirement that genetic instruments exhibit no horizontal pleiotropy yet still produce causal effect estimates that are asymptotically consistent. These approaches often rely on different sets of assumptions to each other, meaning that if the results from all of these different analyses are largely consistent, then the investigator can be more confident in drawing conclusions regarding causality.

MR-Egger regression [42••] is one such approach where given a set of genetic variants that proxy an exposure variable of interest, estimates of the SNP-outcome association are regressed on estimates of the SNP-exposure association (this can be done in a one or two-sample MR framework), where each data point is weighted by the precision of the SNP-outcome coefficients. The slope of the weighted regression is an estimate of the causal effect of the exposure on the outcome. The intercept in this regression is free to vary, and the degree to which it departs from zero, is a function of the degree of directional pleiotropy present in the data.

The MR-Egger approach relaxes the requirement of no horizontal pleiotropy among the SNPs. Instead it assumes that there is no correlation between the gene-exposure association and the direct effect of the genetic variants on the outcome. This is referred to as the InSIDE assumption (Instrument Strength Independent of Direct Effect) and is a weaker requirement than the stricter exclusion restriction criterion. A drawback of the MR-Egger method is that it tends to suffer from low statistical power and is particularly susceptible to bias from weak instruments.

The weighted median estimator [94•] is a complementary method that permits up to 50% of the information in the MR analysis to come from SNPs that are invalid instruments. The mode-based estimate (MBE) further relaxes the assumption required for the weighted median approach and can estimate the causal effect when the most common pleiotropy value across instruments is zero [80•]. In addition, Bayesian modeling alternatives, such as Bayesian model averaging [81•], are under development, and may provide a framework to model pleiotropic effects and further relax MR assumptions, extending the scope of MR analysis.

In some circumstances, effect estimates are not consistent across independent instruments (e.g., with some genetic instruments showing unexpectedly large or small effects on the outcome, given the magnitude of their exposure effect), which could be indicative of horizontal pleiotropy. Formal statistical tests for heterogeneity can be used to assess this, such as Cochran’s Q statistic (for IVW) and the Rucker’s Q (for MR-Egger) [98, 103].

In addition to the above-mentioned methods, visual inspection can be helpful to identify pleiotropic variants (e.g., outlier detection). For example, funnel plots are used to display the MR estimate of individual genetic variants against their precision. Asymmetry in the funnel plot may arise due to some genetic variants having unusually strong effects on the outcome, which is indicative of directional pleiotropy [42••]. In addition, heterogeneous effects can be visualized by scatterplots of the gene-outcome and gene-exposure associations [94•] and forest plots of Wald ratios for each independent genetic instrument. In the leave-one-out plot, one SNP is removed at a time and the overall effect estimate is recalculated so that influential individual SNPs can be identified. As sensitivity analyses, all the above visualization methods are implemented in the MR-Base R package [55••]. Other graphical approaches have been proposed recently, such as the radial plot [99] and Q-contribution plots [103], which can further help to assess heterogeneity across genetic variants and detection of pleiotropic variants.

### The Development of Approaches to Assess Instrument Strength

It is important to assess the instrument strength in order to avoid weak instrument bias in MR analysis. When weak instruments are estimated in GWAS with small sample sizes, MR approaches can violate the “NO Measurement Error” (NOME) assumption, which assume that the SNP-exposure associations (weights of the regression) are estimated without measurement error [43•]. For IVW, weak instruments that violate the NOME assumption can be reliably detected using the mean F-statistic [102]. For MR-Egger, the degree of violation of the NOME assumption can be quantified using the I^2^ statistic (I_GX_^2^), a number ranging between 0 and 1, with higher values indicating less dilution of the causal effect estimate [43•].

## Conclusion

MR is a flexible and robust statistical method which uses genetic variants as instrumental variables to detect and quantify causal relationships in observational epidemiological studies. In this review, we have endeavored to illustrate promising new findings and potential pitfalls of MR. The design strategies, assumptions, limitations, and potential of MR have been discussed. Given the growing availability of large-scale genetic resources and automated toolkits for implementing these methods, such as MR-Base and LD hub, we are now able to analyze all pairwise relationships within large multidimensional data sets in a hypothesis-free manner, producing evidence that can then be followed up in subsequent in-depth investigations.
